# Calcium Apatite Deposition Disease: Diagnosis and Treatment

**DOI:** 10.1155/2016/4801474

**Published:** 2016-11-30

**Authors:** Nicholas M. Beckmann

**Affiliations:** Department of Diagnostic and Interventional Imaging, UT Health, Houston, TX, USA

## Abstract

Calcium apatite deposition disease (CADD) is a common entity characterized by deposition of calcium apatite crystals within and around connective tissues, usually in a periarticular location. CADD most frequently involves the rotator cuff. However, it can theoretically occur in almost any location in the musculoskeletal system, and many different locations of CADD have been described. When CADD presents in an unexpected location it can pose a diagnostic challenge, particularly when associated with pain or swelling, and can be confused with other pathologic processes, such as infection or malignancy. However, CADD has typical imaging characteristics that usually allows for a correct diagnosis to be made without additional imaging or laboratory workup, even when presenting in unusual locations. This is a review of the common and uncommon presentations of CADD in the appendicular and axial skeleton as well as an updated review of pathophysiology of CADD and current treatments.

## 1. Overview

Calcium apatite deposition disease has been given many names (calcific periarthritis, calcific bursitis, periarthritis calcarea, periarthritis calcarea, and hydroxyapatite rheumatism) but is most commonly known as calcific tendinitis. It is a relatively common phenomenon characterized by formation of calcium deposits both in and around tendons and other connective tissue structures. Calcific tendinitis most commonly occurs in between the ages of 30–60, although cases have been described in patients as young as 3 years old [1, 2]. A large majority of cases of calcific tendinitis occur at the shoulder, particularly involving the supraspinatus and infraspinatus tendons. The hip is the next most common location of involvement, followed by the spine. Although rarely reported, calcific tendinitis has been described involving numerous other sites as well [3–6].

Historically, calcium deposits in calcific tendinitis have been thought to represent collections of hydroxyapatite crystals leading to the used of the term hydroxyapatite deposition disease (HADD) to describe this disease process [7, 8]. However, more recent studies by Hamada et al. in 2001 and 2006 demonstrated the calcium deposits of calcific tendinitis to be composed of carbonate apatite instead of hydroxyapatite as previously thought [9, 10]. The exact etiology of these deposits is unknown. Several authors have speculated that the formation may be the result of tendon degeneration, while others have proposed that the calcifications are a cell-mediated reactive process [11–14]. Uhthoff and Loebr proposed a natural progression of calcific tendinitis that can be divided into four phases: precalcific, formative, resorptive, and postcalcific [15]. In the precalcific phase, collagen fibers of the tendon undergo metaplasia into fibrocartilage tissue. During the formative phase, chondrocytes begin to develop within the areas of fibrocartilage formation with eventual formation of calcified apatite crystals. After the formative phase, the area of calcification can remain asymptomatic in a “resting state” for an indefinite period of time. Typically, however, the calcifications will progress to an inflammatory resorptive phase characterized by the appearance of leukocytes, lymphocytes, and giant cells forming a “calcium granuloma.” Finally, the calcification will enter the postcalcific phase where a reparative process incites new capillary and collagen fiber formation.

Calcific tendinitis typically presents with a single site of involvement. However, bilateral presentation of shoulder calcific tendinosis is not uncommon, occurring in anywhere from 5 to 23% of patients in larger studies of rotator cuff calcific tendinitis [16–18]. Bilateral involvement has been rarely described in the hip [19], and bilateral involvement has not been described in any other joint. There is a slight female predilection for calcific tendinitis with calcific tendinitis occurring approximately 50% more frequently in women than men [2]. A study by Harvie et al. showed an association between estrogen and thyroid hormone disorders and the development of calcific tendinitis, which may account for at least a portion of the female predilection [17]. Sengar et al. discovered an association between the HLA-A1 gene and calcific tendinitis, suggesting a genetic predisposition to the disease [20]. An association has also been found between adult onset diabetes and calcific tendinitis [21].

## 2. Clinical Presentation

Calcium apatite deposition is often seen incidentally on radiographs in asymptomatic patients. When patients are symptomatic, symptoms can vary from an acute episode of severe pain to chronic mild discomfort. Typically, acute episodes of pain are single occurrences that resolve spontaneously, although patients can experience recurrence of pain months after the initial episode [22]. Occasionally, patients will present with symptoms of neuropathy [5, 6, 23]. These acute episodes of pain are what frequently lead to patients presenting for treatment and can be a diagnostic challenge for clinicians. Acute episodes of calcific tendinitis frequently present with severe pain along with mild swelling and warmth of the affected tissue, which can be easily mistaken for infection [24, 25]. Restricted range of motion may or may not be present. Laboratory and vital signs are usually normal; however, it is not uncommon for patients to have mildly elevated inflammatory markers, mild leukocytosis, or low grade fever, which can also raise the concern for infection [4, 25–27]. Furthermore, it is not uncommon for acute calcific tendinitis episodes to be preceded by low level trauma, and the amorphous calcifications may be mistaken for a neoplastic process [4, 24, 26].

## 3. Imaging

**Figure 1:**
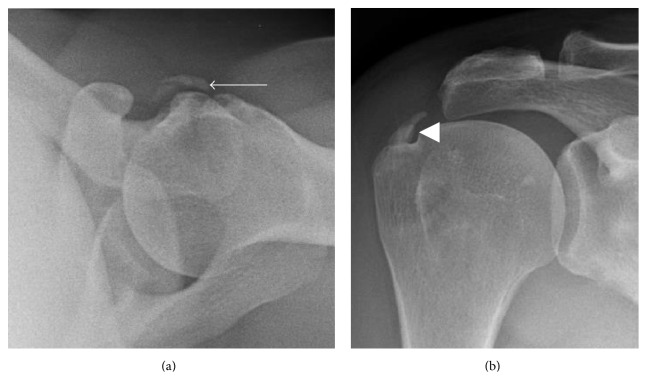
Radiographic appearance of calcific tendinopathy. (a) Fluffy, ill-defined, and inhomogeneous appearance of calcifications (arrow) typical seen in acute symptomatic patients. (b) Discrete, homogeneous, and well-defined appearance of calcifications (arrowhead) typically seen in asymptomatic or chronic symptomatic patients.

**Figure 2:**
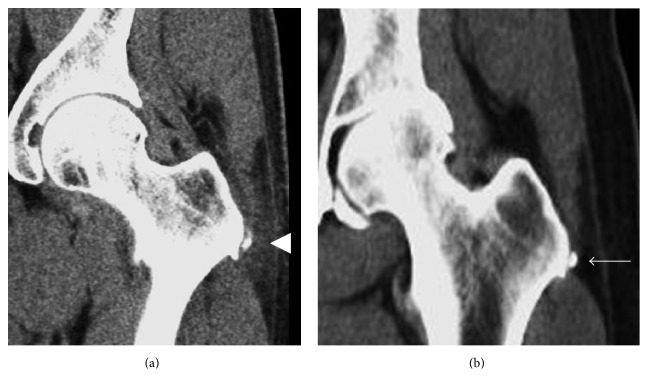
CT appearance of calcific tendinitis. (a) Acute symptomatic phase mirrors radiographic findings with ill-defined, amorphous calcification and associated soft tissue edema (arrowhead). (b) Calcification becomes better defined with resolution of soft tissue edema as the calcific tendinopathy progresses to the chronic phase (arrow).

The diagnosis of calcific tendinitis is most frequently made on radiographs. Calcifications have been described typically appearing as fluffy, ill-defined, and inhomogeneous or as discrete, homogeneous, and well defined (Figure 1) [8, 15, 28]. The fluffy, ill-defined calcifications are associated with the acutely symptomatic phase of calcific tendinitis, while the better defined, homogeneous calcifications tend to be present in patients that are asymptomatic or have chronic pain [15, 28]. Computerized tomography (CT) is not commonly done for the diagnosis of calcific tendinitis, although it has been frequently described in assessment of patients presenting with longus colli calcific tendinitis to exclude traumatic injury or deep soft tissue infection. When it is performed, the appearance of calcification mirrors the appearance of calcification on radiographs (Figure 2), and soft tissue edema may be better visualized on CT.

**Figure 3:**
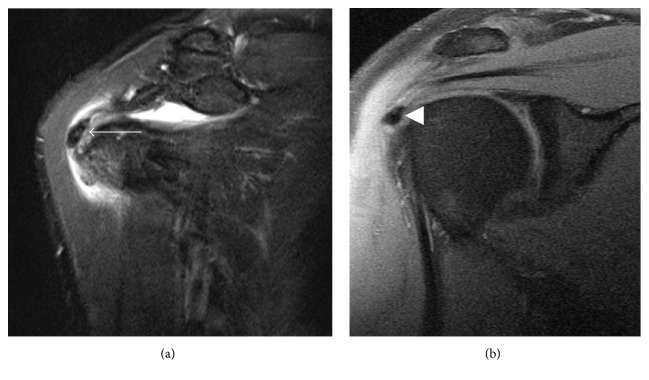
MRI appearance of calcific tendinopathy. (a) The acute, symptomatic phase of calcific tendinopathy is often associated with soft tissue edema surrounding the tendon calcifications (arrow). (b) In the chronic phase of calcific tendinopathy, calcifications are typically more well defined and homogeneously low in signal intensity without surrounding edema (arrowhead).

After radiographs, magnetic resonance imaging (MRI) is the most common modality used to diagnosis calcific tendinitis, as it confirms the presence of inflammation associated with the acute symptomatic phase of calcific tendinitis and excludes other etiologies of pain when the presenting symptoms are atypical. MRI appearance of the calcifications varies depending on the radiographic appearance of the calcifications, and the degree of soft tissue inflammation depends on the stage of the calcifications. In the acutely symptomatic phase, soft tissue edema will typically be seen surrounding the region of calcification (Figure 3(a)) [4, 15, 29]. Discrete, homogeneous calcifications on radiographs appear as homogeneous signal voids on all MRI sequences (Figure 3(b)), while ill-defined inhomogeneous calcifications on radiographs appear as heterogeneous low and intermediate signal on both T1 and fluid sensitive MRI sequences (Figure 3(a)).

**Figure 4:**
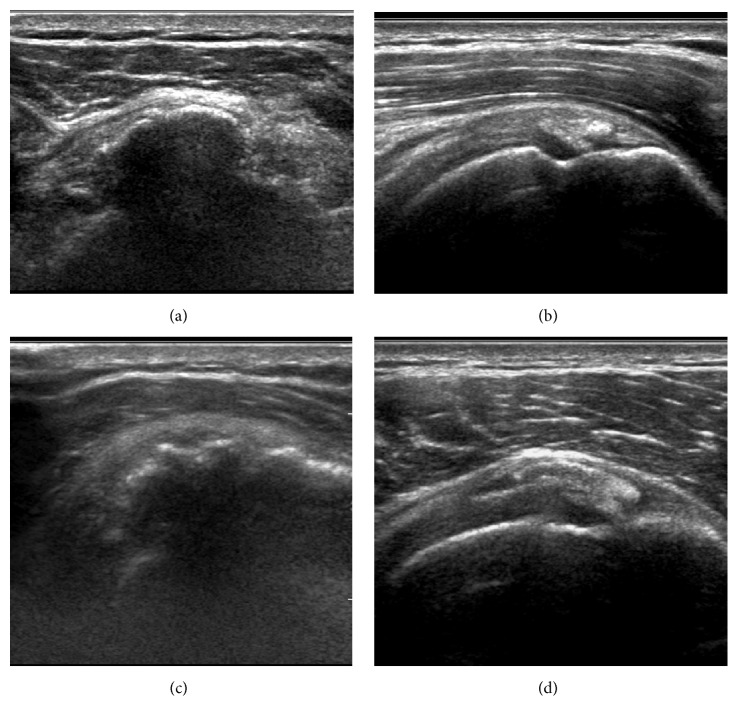
Ultrasound appearance of calcific tendinopathy. (a) Arc morphology: echogenic arc of calcification with deep acoustic shadowing. (b) Nodular morphology: single echogenic focus of calcification without acoustic shadowing. (c) and (d) Fragmented morphology: two or more echogenic foci of calcification with (image (c)) or without (image (d)) acoustic shadowing.

Ultrasound is not commonly needed to make the diagnosis of calcific tendinitis, but it is commonly used in image-guided treatment of the calcifications. Four morphologies of tendon calcification have been described on ultrasound (Figure 4): arc-shaped (echogenic arc of calcification with deep acoustic shadowing), nodular (single echogenic focus of calcification without acoustic shadowing), fragmented (two or more echogenic foci of calcification with or without acoustic shadowing), and cystic (hyperechoic wall with anechoic region, weakly hypoechoic region, or layering content) [30]. The nodular, fragmented, and cystic morphologies on ultrasound are associated with the acute, symptomatic phase of calcific tendinitis while the arc morphology is more suggestive of the chronic or asymptomatic phase [30]. Increased flow on power Doppler is strongly associated with acute symptomatic calcific tendinopathy but is present in only about one-third of cases [31]. In the rotator cuff, thickening of the subacromial-subdeltoid bursa is strongly associated with acute symptomatic calcific tendinopathy but is present in less than one-third of cases [31].

**Figure 5:**
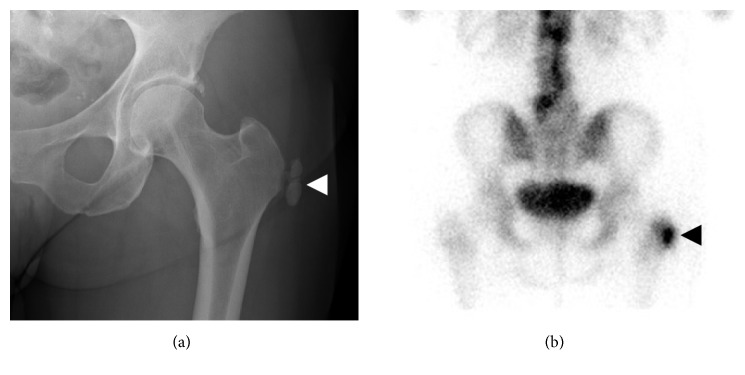
Appearance of calcific bursitis on technetium-99m bone scan. (a) A large conglomeration of soft tissue calcifications reside in the region of the left greater trochanter bursa on AP hip radiograph (white arrowhead). (b) The calcifications demonstrate exuberant increased radiotracer uptake on the delayed phase of technetium-99m MDP bone scan (black arrowhead).

Nuclear medicine exams are rarely used to diagnose calcific tendinitis. Not surprisingly, cases of calcific tendinitis have been described as demonstrating increased radiotracer activity on both positron emission tomography and technetium-99m bone scans (Figure 5) [32–34]. This increased activity is likely related to the inflammatory phase of calcific tendinitis and can be confused for malignancy or bony metastatic disease.

**Figure 6:**
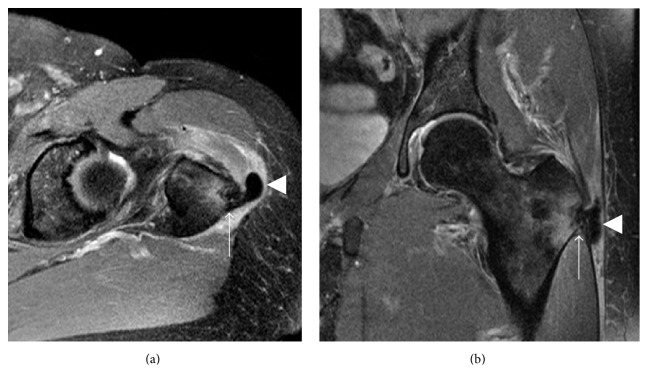
Calcific bursitis with osseous erosion. (a) and (b) Axial and coronal MRI images show a large conglomeration of soft tissue calcification within the greater trochanter bursa with surrounding soft tissue edema (arrowheads). The calcifications about the greater trochanter and erode the cortex with reactive edema of the adjacent bone marrow (arrows).

Rarely, calcific tendinitis can extend to involve the underlying bone at the tendon attachment site. Approximately 80% of osseous involvement by calcific tendinitis occurs at the proximal femur and proximal humerus with the femur diaphysis most commonly involved followed by the humerus tuberosities. Cortical erosion (Figure 6) is the most common presentation of osseous involvement, seen in approximately three-quarters of patients. Periosteal reaction and bone marrow involvement are present in about one-third of patients with intraosseous calcific tendinitis. Periosteal reaction is usually solid and benign in appearance, although it can have an aggressive, lamellated appearance in about one-third of patients [34].

## 4. Locations

### 4.1. Spine

**Figure 7:**
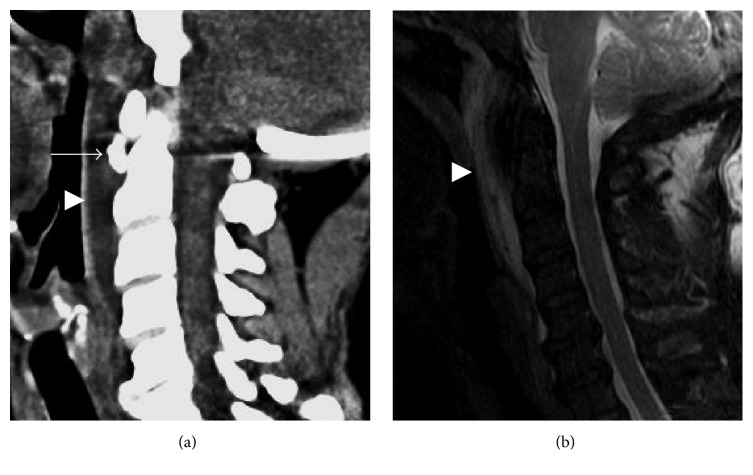
Calcific tendinitis of the longus colli. (a) Sagittal CT showing rounded calcification anterior to the dens just below the C1 anterior arch (arrow). Prevertebral edema causes anterior bowing of the pharyngeal mucosa (arrowhead). (b) Prevertebral edema is more conspicuous on sagittal T2 fat-suppressed image (arrowhead). The calcification of the longus colli tendon is not well appreciated on MRI.

Spine calcific tendinitis has almost been exclusively described to involve the upper oblique fibers of the longus colli tendon at the C1-C2 level (Figure 7). A few case reports have described calcific tendinitis involving the mid to lower longus colli at the C5–C7 levels, and there has been a single case report of involvement of the presacral space [35–37].

Calcific tendinitis of the spine is much less common than involvement of the shoulder. A study by Horowitz et al. estimated the annual incidence of symptomatic longus colli calcific tendinitis at 0.5 cases per 100,000 people [38]. Neck pain is the most common presenting symptom with decreased range of motion, neck stiffness, odynophagia, and dysphagia also being common [38, 39]. Rarely, a patient may have evidence of airway compromise secondary to swelling [36]. Patients typically present within a few days of symptom onset, although subacute symptomatic and chronic asymptomatic cases have been described [39, 40]. Reports of longus colli calcific tendinitis frequently describe leukocytosis, elevated inflammatory markers, and/or low grade fevers at presentation, more so than calcific tendinitis in other parts of the body. However, this may be related to the calcific tendinitis being in a location particularly prone to developing deep soft tissue infections, thereby prompting lab analysis. Symptoms almost always resolve within a few days to weeks following presentation and conservative treatment.

Prevertebral edema is almost always present in symptomatic patients [38, 39]. Calcifications are usually visible on CT or MRI, but they may occasionally be absent [39]. Prevertebral edema is usually confined to the C1–C4 levels but may extend more inferiorly, particularly if the mid to lower longus colli tendon is involved.

### 4.2. Shoulder

**Figure 8:**
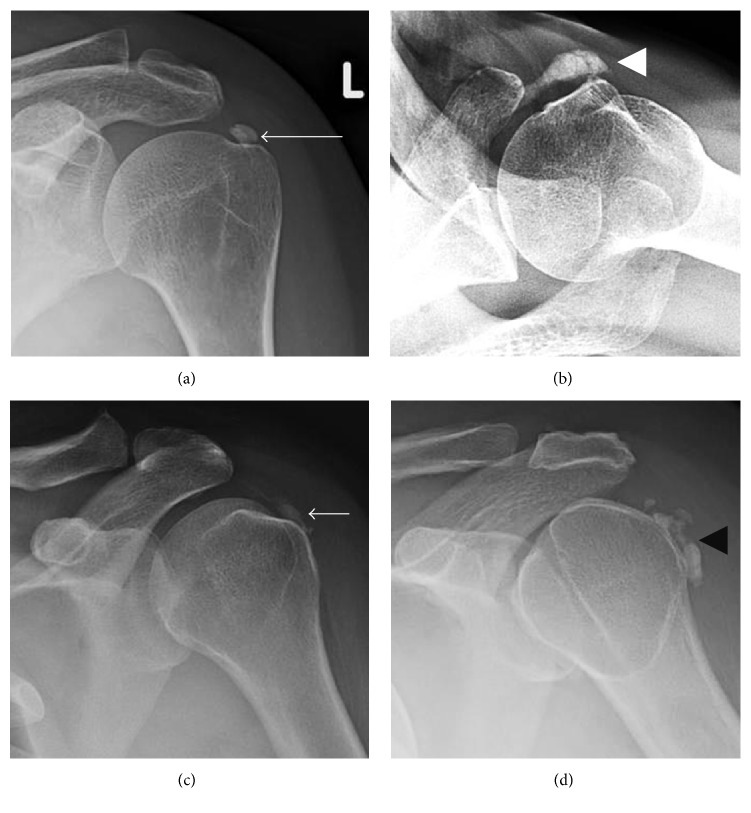
Calcific tendinopathy of the rotator cuff. (a) Calcific tendinopathy of the supraspinatus (long arrow) is best seen on AP shoulder radiograph in external rotation. (b) Calcific tendinopathy of the subscapularis (white arrowhead) is best appreciated on axillary view of the shoulder. (c) and (d) AP views of the shoulder in internal rotation are best for appreciating calcific tendinopathy of the infraspinatus (short arrow) and teres minor (black arrowhead).

The shoulder is the most common location of calcific tendinopathy by a wide margin, with the rotator cuff being the most common area of involvement (Figure 8). The supraspinatus and infraspinatus compose a large majority of rotator cuff cases [41]. The subscapularis is involved in less than 10% of cases and teres minor involvement is even rarer [42–44]. Calcific tendinitis is exceeding common in the shoulder, being present in approximately 3–8% of asymptomatic shoulders and 33–42% of shoulders with symptoms of subacromial pain syndrome [2, 42, 45–47].

The association between rotator cuff calcific tendinopathy and rotator cuff tear is controversial. Jim et al. described seeing rotator cuff tears on arthrography in 28% of patients with calcific tendinopathy, and a study of 74 rotator cuff tears by Wolfgang found tendon calcification in 23% of patients with rotator cuff tears [44, 48]. However, operative studies by McLaughlin and Asherman and Friedman found rotator cuff tears to occur only rarely in the setting of calcific tendinopathy, and an ultrasound study by Chiou et al. of 94 patients with rotator cuff calcific tendinopathy found no cases of rotator cuff tear associated with the region of calcification [45, 49, 50].

**Figure 9:**
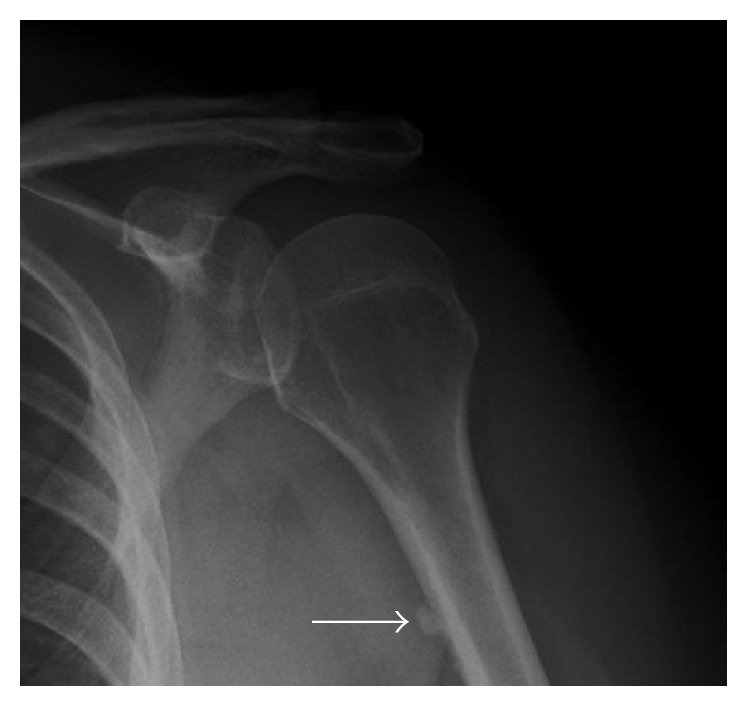
Calcific tendinopathy of the pectoralis major. Pectoralis major calcific tendinopathy can be seen along the anterior cortex of the proximal humerus diaphysis on lateral humerus or internal rotation AP shoulder radiographs (arrow).

Although rare, calcific tendinitis is also known to involve the long head of the biceps tendon and pectoris major insertion (Figure 9). Calcific tendinitis of the long head of the biceps may occur either adjacent to the glenoid at the biceps-labral complex or within the tendon sheath at or distal to the bicipital groove [51–53]. Calcific tendinitis of the long head of the biceps most commonly presents with shoulder pain radiating to the humerus along the course of the biceps tendon [53]. However, it can present with subacromial pain simulating subacromial impingement or rotator cuff tear and can be mistaken for symptoms of a septic glenohumeral joint in a patient with acute symptoms [51, 53].

Pectoralis major calcific tendinitis typically presents focal tenderness over the anterior aspect of the proximal third of the humerus at the attachment site of the pectoris major tendon [54, 55]. The pain is often focal but can radiate down the arm, and patients may have increased pain with shoulder adduction or internal-external rotation. Calcification of the pectoralis major tendon usually occurs at the tendon insertion on the humerus, and erosion of the adjacent cortex is a frequently described associated finding [55–57].

### 4.3. Elbow

**Figure 10:**
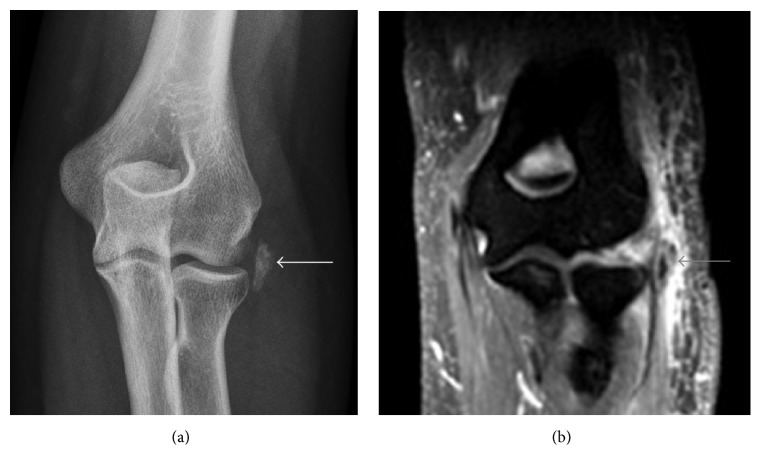
Calcific tendinitis of the common extensor tendon origin. (a) AP radiograph shows amorphous calcifications along the lateral aspect of the elbow joint (white arrow). (b) Exuberant soft tissue edema can be seen around the calcifications, which are centered within the common extensor tendon (grey arrow).

The elbow is the least common joint described to be involved in calcium apatite deposition, although this is likely due to underreporting as calcific tendinopathy of the distal biceps and common extensor/flexor tendons is not an uncommon finding on radiographs of the elbow. Most commonly, calcific tendinitis has been described involving the distal biceps tendon [1, 5, 58, 59]. Patients with distal biceps involvement typical present with focal tenderness over the proximal radius with limited pronation-supination and preserved elbow flexion. Calcific tendinitis of the common extensor (Figure 10) and common flexor tendons has typically presents with symptoms mimicking common extensor or common flexor tendinitis [60, 61].

### 4.4. Hand/Wrist

**Figure 11:**
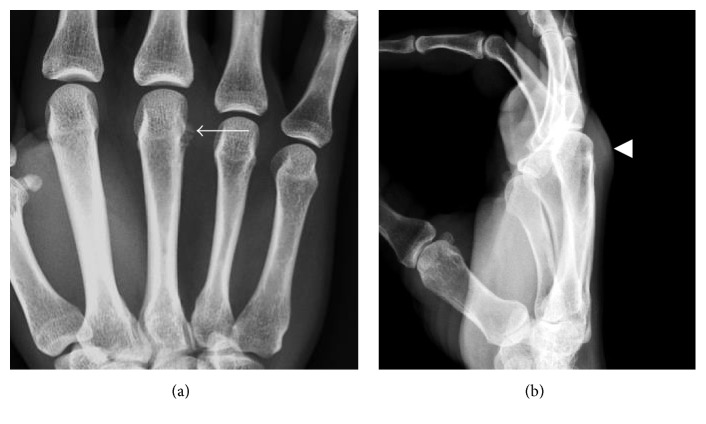
Calcific periarthritis of the third metacarpophalangeal joint. (a) Small amorphous capsular calcifications are present along the ulnar aspect of the third metacarpal neck (arrow). (b) Associated soft tissue swelling of the third metacarpophalangeal joint is present, best appreciated on a lateral view (arrowhead).

**Figure 12:**
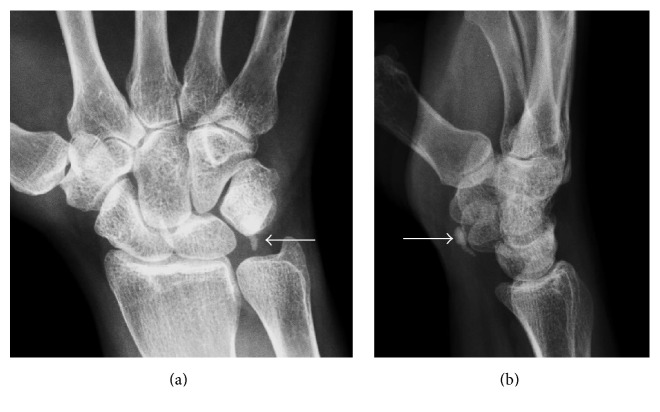
Calcific tendinopathy of the flexor carpi ulnaris. (a) and (b) Small conglomeration of well-defined calcifications are present just proximal to the pisiform in the expect region of the flexor carpi ulnaris insertion (arrows).

Hand and wrist involvement by calcium apatite deposition disease has been well described in the literature. Calcium apatite can present as calcific periarthritis involving the interphalangeal or metacarpal phalangeal joints (Figure 11) or as calcific tendinitis, most commonly involving the flexor carpi ulnaris (Figure 12) with involvement of the flexor digitorum, extensor pollicis longus, flexor pollicis longus, and abductor pollicis brevis also being described [3, 62–65]. As in other parts of the body, CADD of the hand and wrist typically presents as focal tenderness and swelling with or without decreased range of motion of the involved tendon or joint. Occasionally, CADD may involve the flexor digitorum tendons within the carpal tunnel leading to symptoms of carpal tunnel syndrome secondary to median nerve compression [64]. Trigger finger symptoms have also been described in patients presenting with CADD of the flexor digitorum tendons more distally at the level of the metacarpophalangeal joint [66]. The age distribution of CADD in the hand and wrist is similar to CADD in other parts of the body, although a study by Kim and Park of 30 patients with CADD the noted an average age of 45 years in patients with calcific tendinitis of the hand, which was significantly higher than the average age of 35 years in patients with calcific periarthritis [3].

### 4.5. Hip

**Figure 13:**
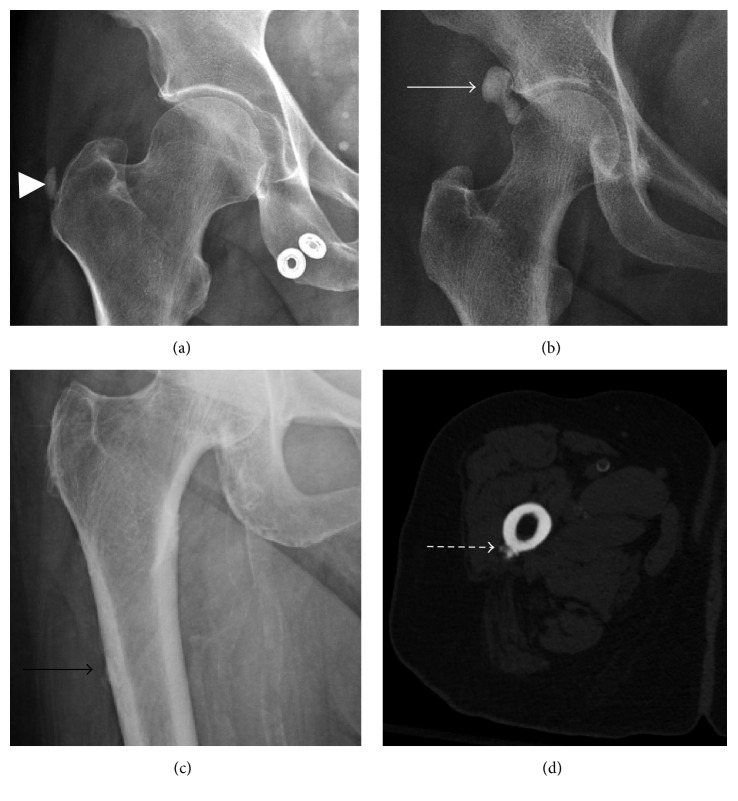
Calcific tendinopathy of the hip. (a) Gluteus medius/minimus calcific tendinopathy (arrow head) is best appreciated on AP hip or pelvis radiographs. (b) Rectus femoris calcific tendinopathy most commonly presents lateral to the acetabular weight-bearing surface (solid arrow). (c) and (d) Calcific tendinopathy of the gluteus maximus tendon. The gluteus maximus tendon calcification almost always involves the tendon insertion on the gluteal tubercle along the posterolateral subtrochanteric femur (black arrow). Cortical erosion and periosteal reaction are frequently seen (dashed arrow), which can easily be mistaken for a juxtacortical malignancy.

After the shoulder, the hip is the most commonly involved region for calcific tendinitis [19]. The gluteus medius (Figure 13(a)), rectus femoris (Figure 13(b)), and gluteus maximus (Figures 13(c) and 13(d)) are well-described areas of involvement. Gluteus maximus calcific tendinitis typically presents with pain over the posterior aspect of the proximal thigh [67, 68]. Patients may have limited abduction-adduction of the thigh. The calcification almost invariably involves the gluteus maximus insertion on the gluteal tubercle along the posterolateral subtrochanteric femur [67, 69]. Cortical erosion of the proximal femur is a commonly described finding and can be mistaken for malignancy [69, 70].

Rectus femoris calcific tendinitis typically presents as focal tenderness over the anterior-inferior iliac spine and limited flexion of the hip [71]. Symptoms can be quite severe and confused for labral tear or septic hip joint [19, 72, 73]. Pierannunzii et al. described a single case of rectus femoris calcific tendinitis resulting in symptoms of a snapping hip [74]. The indirect head of the rectus femoris has been described to be involved up to 9 times more frequently than the direct head [19].

Gluteus medius calcific tendinitis occurs at the gluteal tendon attachments to the greater trochanter [27, 75]. Calcifications may be found in the greater trochanter bursa adjacent to the gluteal tendons, which are sometimes referred to a calcific trochanteric bursitis, although the clinical presentation is identical to gluteus medius calcific tendinitis [76]. Calcifications that occur over the superoposterior portion of the greater trochanter have been described as being associated with lower back, buttock, or posterolateral thigh pain while calcifications centered over the lateral aspect of the greater trochanter have been associated with anterolateral thigh or groin pain [27].

**Figure 14:**
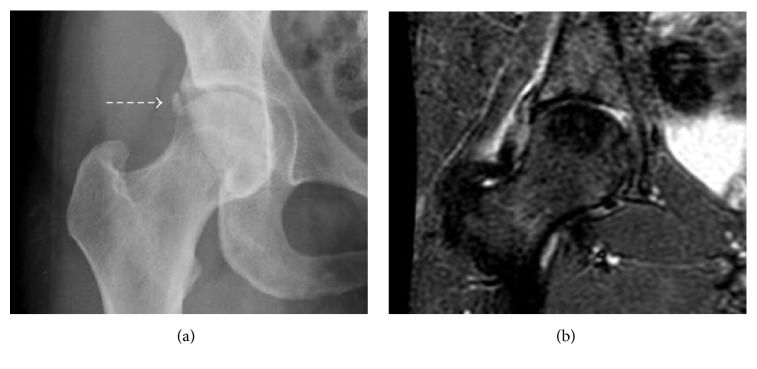
Calcific periarthritis of the hip. (a) and (b) Calcific periarthritis of the hip (dash arrow) presents as periarticular calcifications. In the inflammatory phase of the disease, associated soft tissue edema (image (b)) can help differentiate calcific periarthritis from an os acetabuli.

Other regions of calcific tendinitis around the hip have been described, including distal psoas tendon, joint capsule (Figure 14), ligament teres, adductor magnus, and piriformis [19, 77–81]. However, reports of these locations are sparse with only a few cases in the literature.

### 4.6. Knee

**Figure 15:**
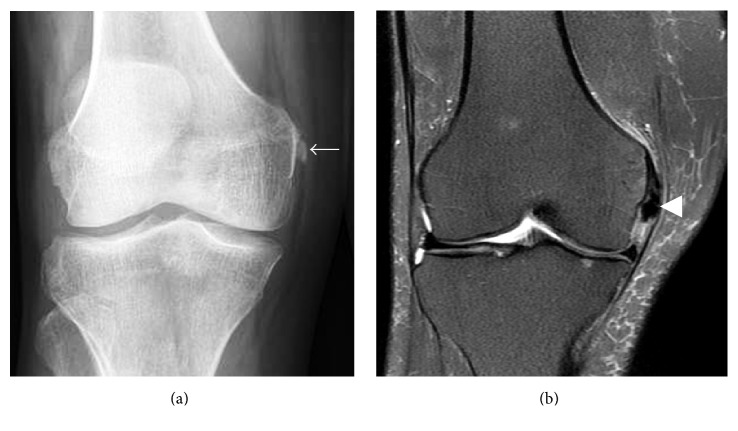
Calcific periarthritis of the proximal medial collateral ligament. (a) Rounded, amorphous calcification adjacent to the adductor tubercle (short arrow). (b) Calcifications (arrowhead) lie deep to the origin of the superficial band of the medial collateral ligament with mild adjacent soft tissue and minimal adjacent bone marrow edema.

Calcific tendinitis of the knee is rare with only a few case reports of knee involvement in the literature. Calcific tendinitis/periarthritis has been described to involve the lateral collateral, medial collateral (Figure 15), and anterior cruciate ligaments as well at the popliteus, quadriceps, medial head of the gastrocnemius, and biceps femoris tendons and prepatellar bursa [73, 81–86]. As in other cases of calcium apatite deposition, patients typically present with focal tenderness over the region of calcification and may have limited range of motion. The reported age range is similar to calcium apatite deposition in other parts of the body with most patients being young adult to middle aged. Calcific periarthritis of the medial and lateral collateral ligaments typically involves the femoral attachment sites [83, 84, 87]. Similarly, popliteus calcific tendinitis tends to occur at the tendon insertion on the lateral condyle of the femur [85, 88]. Calcific tendinitis of the quadriceps can occur at the quadriceps insertion on the superior patellar pole [81, 89].

### 4.7. Foot/Ankle

**Figure 16:**
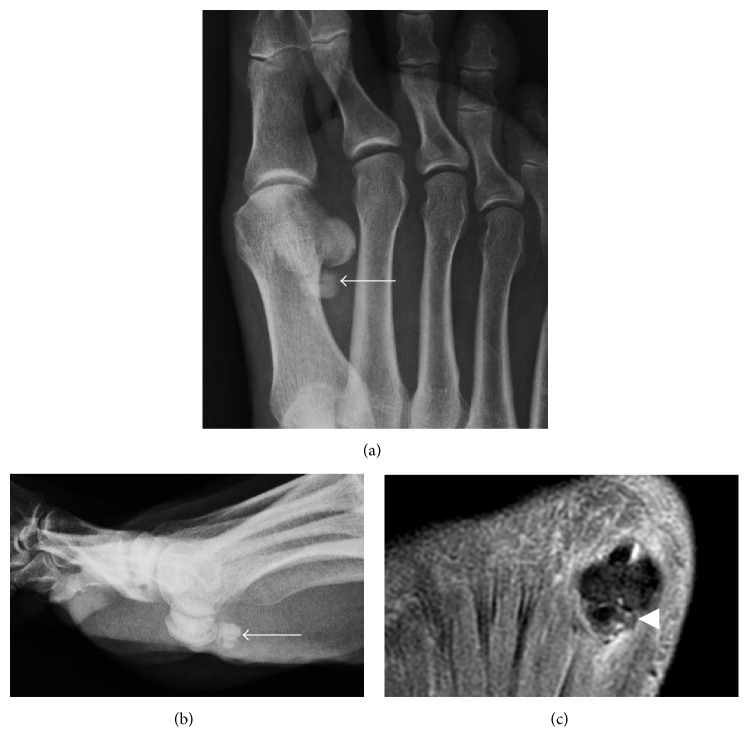
Calcific tendinitis of the flexor hallucis brevis. (a) and (b) Amorphous calcification consistent with acute, symptomatic phase of calcific tendinitis is present just proximal to the hallucal sesamoids (arrows). (c) Long axis PD fat-saturated MR image shows heterogenous signal of the calcification (arrowhead) with mild surrounding edema, which is also consistent with acute symptomatic phase.

CADD involvement of the foot and ankle has only been sparsely reported in the literature. The most commonly described area of involvement is the great toe, either presenting medial to the first metatarsophalangeal joint or at the flexor hallucis brevis tendon just proximal to the hallucal sesamoids (Figure 16) [4, 90]. When CADD involves the flexor hallucis brevis, it typically presents with focal tenderness along the plantar forefoot near the first metatarsal head and can be mistaken for hallucal sesamoiditis [4]. CADD of the first metatarsophalangeal joint can mimic gout arthropathy. However, slightly younger age distribution, female gender predilection, and normal serum urate levels can help distinguish calcium deposits of CADD from gouty tophus [90]. Less common sites of involvement described include the peroneus longus at the peroneal groove, navicular insertion of the tibialis posterior, and second through fifth metatarsophalangeal joints [90–92].

## 5. Differential Diagnosis

**Figure 17:**
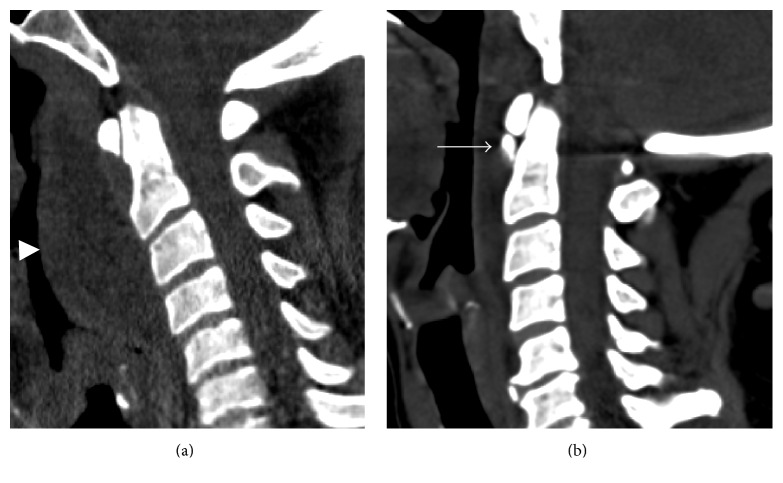
Calcific tendinitis mimicking infection. (a) Marked retropharyngeal edema (arrowhead) in a patient with retropharyngeal cellulitis. (b) Similar but less exuberant retropharyngeal edema in a patient with longus colli calcific tendinitis. The calcification of the longus colli (arrow) allows differentiation of calcific tendinitis from infection.

**Figure 18:**
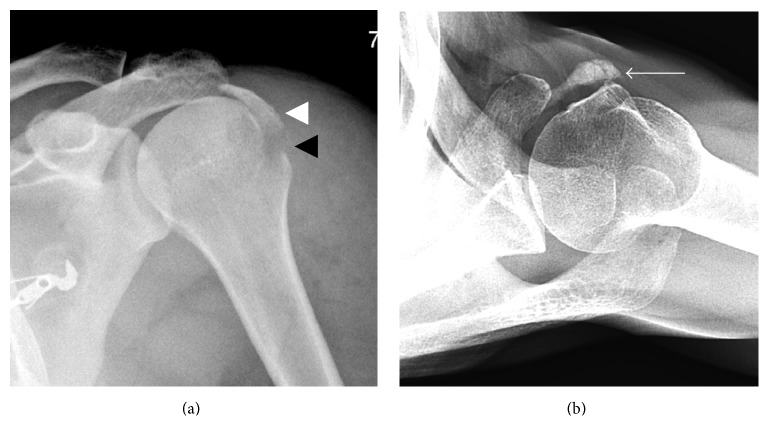
Fracture mimicking calcific tendinitis. (a) A large displaced avulsion fracture of the greater tuberosity is present (white arrowhead). The avulsion somewhat resembles calcific tendinopathy, although incomplete cortication of the avulsion fragment in addition to an adjacent donor site (black arrowhead) allows the diagnosis of avulsion fracture to be made. (b) Calcific tendinopathy of the subscapularis (arrow) resembling an avulsion of the rotator cuff. However, calcific tendinopathy has a rounded and more amorphous appearance of the calcification with no fracture donor site being present.

CADD can be confused with several entities, particularly if a clinician is not aware of the many different locations CADD can present. Acute symptomatic CADD is most commonly mistaken for a traumatic or infectious process, particularly when it occurs in the longus colli of the cervical spine (Figure 17). The presence of the characteristics calcifications is the key element in differentiating CADD from infection. When patients present with acute symptomatic CADD in the setting of minor trauma, the calcifications of CADD may be mistaken for avulsion fragments. However, avulsion fractures should have a linear or incompletely corticated appearance in contrast to the more ill-defined or homogeneous appearance of CADD calcification (Figure 18).

Chronic symptomatic CADD is more commonly confused with either late sequela of trauma (i.e., heterotopic ossification) or with malignancy. Heterotopic ossification can be differentiated from CADD by the presence of a corticated margin around the heterotopic ossification, which is not present in CADD. The bony erosion and periosteal reaction seen in long-standing CADD can make differentiating CADD from a soft tissue malignancy challenging. In this setting, the absence of a discreet soft tissue mass in conjunction with calcification occurring at a typical location of CADD can help distinguish CADD from a neoplastic process.

## 6. Treatment

There is no consensus for appropriate treatment of CADD. Pain in most cases of acute symptomatic calcific tendinopathy will resolve with conservative management, and most calcification will decrease in size or resolve [18]. Cases refractory to conservative management have been treated by a myriad of different methods, including open resection, arthroscopic resection, steroid injection, ultrasound-guided needle lavage (barbotage), extracorporeal shockwave therapy (ESWT), and platelet-rich plasma injection [43, 93–97]. In general, minimally invasive therapies (i.e., ESWT, steroid injection, or barbotage) are first attempted for treatment of calcifications with surgery usually reserved for cases in which minimally invasive techniques are unsuccessful.

There is little quality research comparing effectiveness of different minimally invasive therapies for CADD. A meta-analysis of barbotage studies by Vignesh et al. in 2015 concluded that while there are many low quality studies showing barbotage to be effective in reducing pain and size of calcium deposits, there is only weak evidence that barbotage is more effective than no therapy or needling alone [95]. An additional meta-analysis of minimally invasive therapies by Louwerens et al. in 2014 also came to the conclusion that only low quality evidence exists that barbotage was more effective than no therapy or isolated subacromial steroid injection for treating calcific tendinopathy [93]. Louwerens et al. determined that moderate quality evidence was present showing high-energy ESWT to be more effective than low-energy ESWT or no intervention. However, a recent prospective study by Kim et al. comparing barbotage with ESWT found barbotage to be more efficacious than ESWT in reducing calcification size and improving function and pain relief in the short term [94].

## 7. Conclusion

CADD is a common entity that can present in many different locations. Clinically, CADD can be divided into an acutely symptomatic phase and chronic or asymptomatic phase. Both of the phases have typically imaging characteristics allowing the diagnosis of CADD to confidently be made even when presenting in an atypical location. Acutely symptomatic CADD will usually improve with conservative therapy alone, while chronic CADD may be treated with multiple minimally invasive techniques or surgery. There is conflicting evidence on the best treatment for persistent symptomatic CADD. Both ESWT and barbotage are commonly employed treatment methods that have shown potential for providing symptomatic relief. However, there are few quality studies comparing different treatment methods, and further research is needed to determine optimal management of CADD.
